# A new nanoscale metastable iron phase in carbon steels

**DOI:** 10.1038/srep15331

**Published:** 2015-10-27

**Authors:** Tianwei Liu, Danxia Zhang, Qing Liu, Yanjun Zheng, Yanjing Su, Xinqing Zhao, Jiang Yin, Minghui Song, Dehai Ping

**Affiliations:** 1 Department of Materials Science and Engineering, China University of Petroleum, Beijing 102249, China; 2 School of Materials Science and Engineering, University of Science and Technology Beijing, Beijing 100083, China; 3 School of Materials Science and Engineering, Beihang University, Beijing 100191, China; 4 Jiangsu Asian Star Anchor Chain Co. Ltd., Jingjiang 214533, China; 5 National Institute for Materials Science, Sengen 1-2-1, Tsukuba 305-0047, Japan

## Abstract

Metastable ω phase is common in body-centred cubic (bcc) metals and alloys, including high-alloying steels. Recent theoretical calculations also suggest that the ω structure may act as an intermediate phase for face-centred cubic (fcc)-to-bcc transformation. Thus far, the role of the ω phase played in fcc-bcc martensitic transformation in carbon steels has not been reported. In previous investigations on martensitic carbon steels, extra electron diffraction spots were frequently observed by transmission electron microscopy (TEM), and these spots were historically ascribed to the diffraction arising from either internal twins or carbides. In this paper, an intensive TEM investigation revealed that the extra spots are in fact attributed to the metastable ω phase in particle-like morphology with an overall size of several or dozens of nanometres. The strict orientation relationships between the ω phase and the ferrite matrix are in good agreement with those of the hexagonal (P6/mmm) ω phase in other bcc metals and alloys. The identification of the ω phase as well as the extra diffraction spots might provide a clue to help understand the physical mechanism of martensitic transformation in steels.

It has long been known that the rapid quenching of carbon steels can lead to the formation of a very hard phase, martensite, and its outstanding hardness has been recognized to arise from the supersaturated carbon in ferrite formed by a diffusionless martensitic transformation in which the carbon—formerly in face-centred cubic (fcc) austenite—remains in the bcc martensite. Because of its vital importance to metallurgical engineering and academic research in solid-state physics, the above fcc-to-body-centred cubic (bcc) transformation has received persistent attention since the 1920s, and voluminous experimental work as well as theoretical considerations can be found in the literature^1^,^2^. Nevertheless, because of the complexity of martensitic transformations in steels with different compositions, a convincing theory has not satisfactorily demonstrated the specific transformation process, particularly the mechanism for lattice transition from fcc to bcc. A prevailing crystallographic theory of the martensitic transformation—also called the phenomenological theory—was proposed in the 1950s based on the Bain strain model, and an assumption was made that the interface between austenite and martensite is undistorted at a macroscopic scale. Although the phenomenological theory has been successfully applied to describe the crystallographic characteristics—such as the shape deformation, the orientation of the habit plane and the orientation relationship between parent and product phases^1^,^2^—it is not applicable to elucidating the mechanism underlying the displacement and shuffling of atoms during martensitic transformation in steels. For example, the (225) and (259) habit planes observed in certain high-carbon Fe-C and Fe-Ni-C steels cannot be explained by a single (112) twinning system in martensitic variants predicted by the phenomenological theory^3^,^4^,^5^.

The complexity of martensitic transformation in steels has long been recognized to be closely associated with the existence of carbon because carbon can exert a significant influence on the properties of martensite, such as its hardness, morphologies and substructures. The morphology of the martensites in carbon steels is always observed in two distinctly different types depending on the content of carbon: lath martensite in low-carbon steels and lenticular martensite in high-carbon steels. Twins, often observed as a substructure of martensite in high-carbon steels, were confirmed to be of the {112}<111> type and considered to be similar to other bcc metals and alloys^6^,^7^,^8^,^9^,^10^; dislocations are observed to be characteristic of martensitic substructure in low-carbon steels^3^,^4^,^5^. It is worth noting that during TEM observations, extra diffraction spots at the positions of 1/3{112} and 2/3{112} were invariably observed in the diffraction patterns from the martensitic [110]_bcc_, [113]_bcc_, and [120]_bcc_ zone axes, and these extra diffraction spots were attributed to the double diffraction of the twins in some studies^11^,^12^,^13^,^14^,^15^,^16^. However, the same diffraction patterns with extra spots were also found in martensitic steel comprising lath martensite with dislocations as its substructure. In this case, some researchers considered the extra diffraction spots to have arisen from carbides^17^,^18^,^19^,^20^. Obviously, these different explanations for the same extra diffraction spots in different types of martensitic steels are contradictory. Clarifying the origin of the extra diffraction spots in martensitic steels is helpful to understanding the mechanism of the martensitic formation and involution process.

Very recently, Ping *et al.* noticed that the extra diffraction spots in commercial spring steel with medium carbon content can be indexed by an ω phase^21^ a metastable phase commonly existing in other bcc metals and alloys^22^,^23^,^24^. Theoretical calculations also suggested that carbon is a stabilizing element for the ω phase in steels^21^. Very recent investigations of the phase transition pathway by first-principles calculations showed that the ω structure appears during the fcc-bcc transformation^25^, and the phase transformation follows a route of fcc-ω-bcc or fcc-ω+bcc^25^,^26^. Studies on the martensitic transformation in steels have been conducted for almost one hundred years. If the ω structure is a prior phase to martensite, it must have left traces, whereas current observation and analysis are able to exploit the increased power of modern instrumentation. In the present study, we strictly exclude certain other possible explanations for the extra spots in diffraction patterns, such as double diffraction of twinning or diffraction of carbide, and confirm that the extra spots can be attributed to the ω phase. It is expected that the metastable phase will provide a clue in searching for a physical explanation of the mechanism of martensitic transformation in steels.

## Results

**Figure 1:**
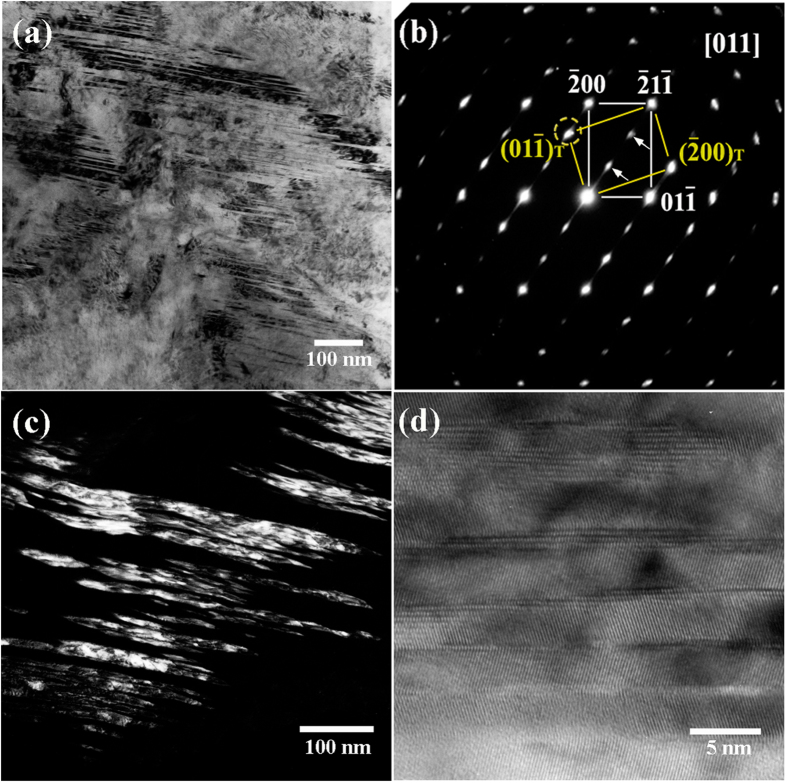
TEM results from the as-quenched Fe-0.58 mass% C sample. (**a**) Bright-field micrograph revealing a high density of twins in the martensite. (**b**) The corresponding SAED pattern with a {112}<111>-type twinning structure observed together with the ω phase. (**c**) The dark-field micrograph imaged using the diffraction spot outlined by the dashed circle in (**b**). (**d**) A high-resolution TEM lattice image of the {112}<111> twins together with the ω phase at the twinning boundaries.

Figure 1(a,b) depict a typical bright-field TEM image and its corresponding selected area electron diffraction (SAED) pattern of martensite with internal twins as a substructure in the as-quenched Fe-0.58 mass% C sample, respectively. The dashed circle in Fig. 1(b) indicates a twin spot in addition to the normal bcc [011] zone diffraction spots, and two arrows indicate extra spots at 1/3(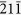) and 2/3(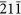) in addition to the matrix and twin diffraction patterns. The dark-field TEM image shown in Fig. 1(c) was captured using the diffraction spot outlined by the dashed circle in Fig. 1(b). It was generally believed that the twin boundary planes in metallic materials should be straight or sharp even at the atomic level^4^,^27^. However, it is interesting to note that the twins or twin boundaries are curved in the dark-field image (Fig. 1(c)) with higher magnification, although they seem straight in the bright image with lower magnification.

Figure 1(d) shows a high-resolution TEM lattice image of the {112}<111> twins in Fig. 1(a). One can see clearly that the width of the matrix is almost identical to that of the twin parts. The twinning boundaries are indeed not sharp at the atomic level, consisting of “interrupted” or “overlapped” atomic layers. Obviously, these interrupted or overlapped twinning boundaries are controversial with the conventional twinning mechanism for martensite—i.e., shear mechanism. On the basis of the conventional twinning mechanism, the twinning boundaries should be atomically sharp and straight if the incident electron beam is parallel with the twinning zone axes, such as the case shown in Fig. 1(b). In the present study, a large number of high-resolution TEM observations on the twinning boundaries were made, and almost all twinning boundaries were not straight at the atomic level.

In addition to the diffraction spots from the matrix and twin crystals, the extra spots—as indicated by arrows in Fig. 1(b)—also exist. In previous studies of martensitic steels, such extra diffraction spots were frequently observed and were usually treated as spots arising from double diffraction by the {112}<111> twinning structure^11^,^12^,^13^,^14^,^15^,^16^. According to electron diffraction theory, double diffraction is a dynamical effect whereby electrons that have been Bragg diffracted by one crystal satisfy the Bragg condition for another^15^. Thus, double diffraction can occur when the electron beam is diffracted by twins in a crystal structure, but does not occur in a single crystal with a disordered cubic structure. Indeed, theoretical treatment of the double diffraction of twinning in bcc and fcc lattices has indicated that double diffraction can occur in {111}_fcc_, {112}_bcc_ systems^14^,^15^. In these theoretical treatments, the matrix is regarded as the first crystal and the twin as the second crystal. Previous studies^13^,^28^,^29^ have explained these extra spots as double diffraction that can occur when the size of the twins is at the nanoscale and the observed region is sufficiently thick to contain several twins in the depth direction. Nonetheless, if the incident electron beam is parallel with the twinning boundary plane and there is no overlap between the matrix and the twin in the depth direction, it is normally impossible to observe any double diffraction in the twinning diffraction pattern.

**Figure 2:**
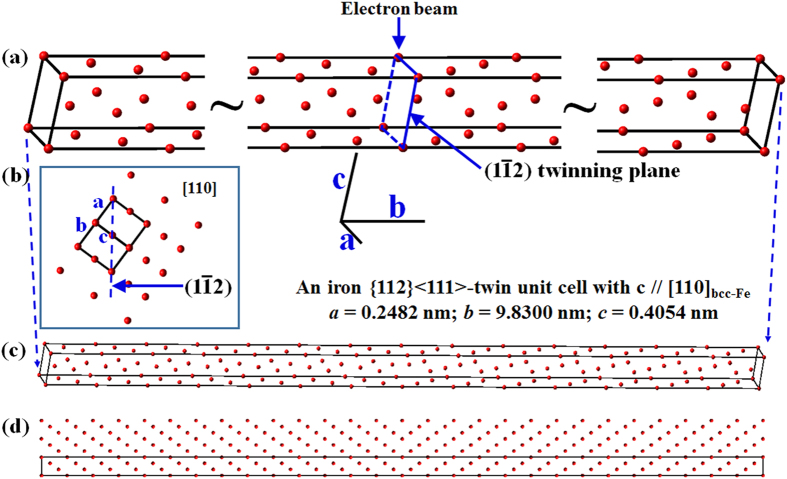
(**a**) Parts of the constructed unit cell; the whole twinning unit cell with *a* = 0.2482 nm, *b* = 9.83 nm and *c* = 0.4054 nm. (**b**) Two-dimensional atom configuration of bcc structure projected along the [110] direction. (**c**) A smaller scale of (**a**) showing all the atoms in one twinning unit cell. The constructed {112}<111>-type twin has 84 atoms, and the twinning plane is in the middle and the end of the cell. (d) The projected atom configuration of three twinning unit cells along [110].

To clarify the dynamic diffraction pattern in a bcc twin system, xHREM (a software for calculating high-resolution TEM image, developed by HREM Research Inc., at Higashimatsuyama 355-0055, Japan) was employed to perform a dynamic calculation on the bcc {112}<111>-type twinning. Figure 2 represents a {112}<111>-type twinning unit cell for the calculation with *a* = 0.2482 nm, *b* = 9.83 nm, *c* = 0.4054 nm and a twinning plane in the middle. Such a unit cell consists of a bcc {112}<111>-type twinning structure. During the calculation, the software generated an arbitrary repeat of the unit cell periodically along its *a* and *b* axes. In this situation, another twinning plane exists at the end of the cell because the unit cell is under a periodic boundary condition. The electron beam is parallel with the *c* axis, and a partial projection along the [110]_bcc-Fe_ direction is shown in Fig. 2(b). All atoms can be seen in a small-scaled map as shown in Fig. 2(c). Figure 2(d) shows three twinning unit cells projected along the [110] direction.

**Figure 3:**
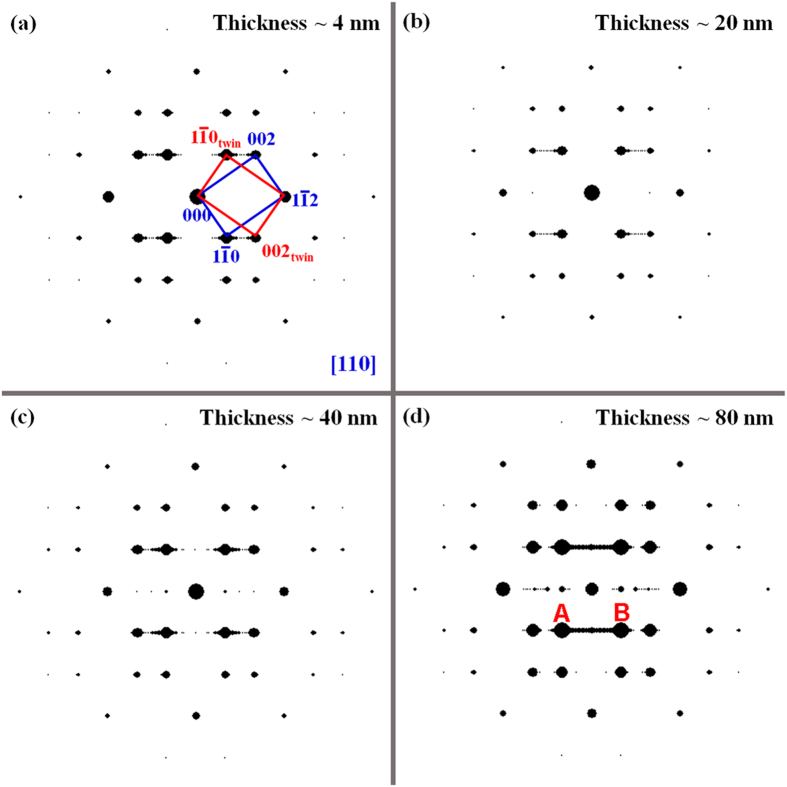
Calculated diffraction patterns of a {112}<111>-type twinning structure with various thickness. The dashed spots along the [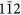] direction are caused by the super-lattice structure of the twinning interface during calculation because the twinning unit cell was repeated many times to increase the thickness and width.

The calculated dynamic twinning diffraction patterns with several different thicknesses along the [110] zone axis are shown in Fig. 3. The twinning unit cell is repeated along [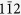] during calculation; therefore, the twinning plane—which can be regarded as “a plane defect”—is also repeated and has a periodic distribution along the same direction. When the thickness is approximately 4 nm, the diffraction spots are in a regular twinning pattern; however, some weak dots or streaking between spots 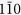
_twin_ and 002 can be seen in Fig. 3(a). These weak spots or streaking are most likely caused by the super-lattice property of the twinning plane constructed in the present calculation. Because these super-lattice spots will change their position if the periodic distance of the constructed twinning plane changes—in reality, the twin plane distance is not constant, varies randomly and thus does not even produce any super-lattice spots—these super-lattice spots appearing in the calculation might not be responsible for the “double diffraction spots”. When the thickness is increased to 80 nm—which is normally a comparable thickness to that in the TEM observation region—a very strong line—which can be many strong spots connected to one another—appears between spots A and B, as shown in Fig. 3(d). Such strong lines have never been observed during experimental observations. From Fig. 3(a) through (d), in addition to these super-lattice spots, the so-called “double diffraction spots” of the {112}<111>-type twinning structure cannot be observed, regardless of the sample thickness. Thus, the interpretation of the extra spots in terms of double diffraction could not provide any evidence from the present dynamic calculation.

**Figure 4:**
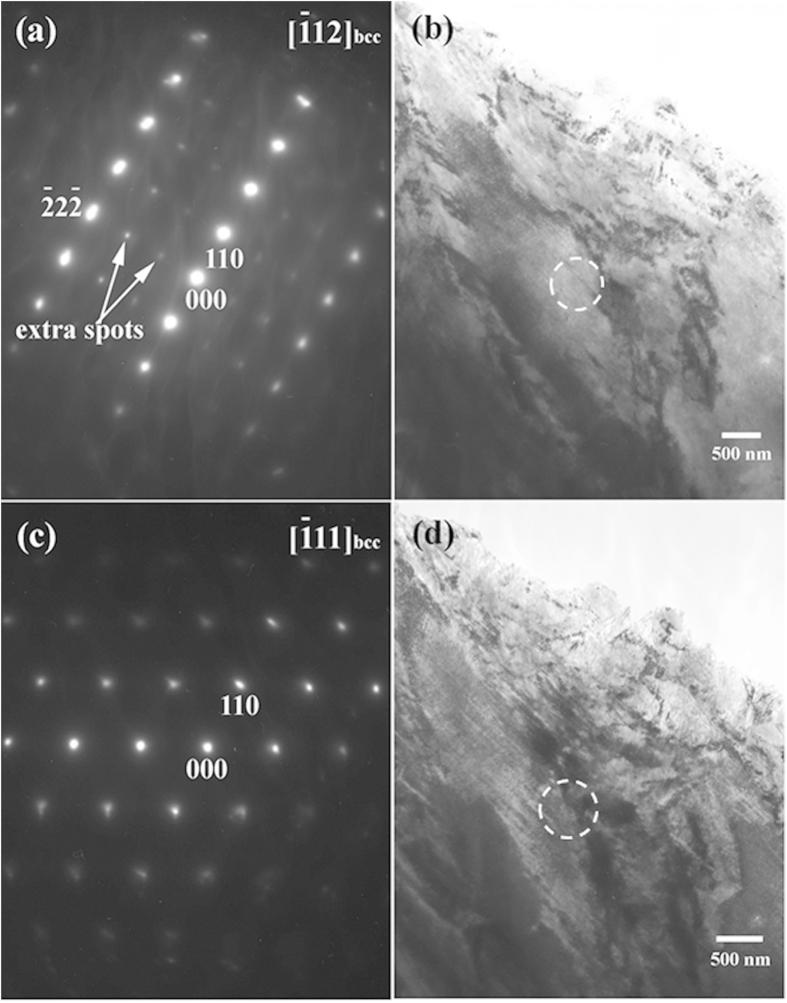
The SAED patterns of [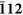]_α_ and [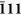]_α_ zone axes and their corresponding bright-field TEM images of the as-quenched Fe-0.58 mass% C sample, respectively. (**a,b**) are the SAED pattern from α-Fe with the [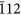]_α_ zone axis parallel to the incident electron beam and the corresponding bright-field TEM image in which the SAED pattern was taken from the circled region. (**c,d**) are the SAED pattern from the [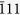]_α_ zone axis after tilting (**a**) approximately 20° and the corresponding bright-field TEM image in which the SAED pattern was taken from the circled region.

For further investigation of the extra spots appearing in the electron diffraction of martensites, diffraction patterns from various zone axes were recorded. Figure 4(a,b) show the SAED pattern of the [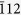]_α_ zone axis and the corresponding bright-field TEM image of the as-quenched Fe-0.58 mass% C sample, respectively. The pattern shown in Fig. 4(a) clearly reveals two sets of diffraction spots. One set with strong spots is from α-ferrite, whereas the other set of weak spots can be seen at the positions of 1/3(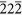) and 2/3(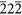). These extra spots cannot be treated as “double diffraction” of the {112}<111>-type twinning structure because the twinning plane is {112}, which is perpendicular to the incident electron beam. In such cases, all matrices and twins will result in exactly the same diffraction spots and the diffraction patterns should be totally overlapped. Obviously, these extra weak diffraction spots must arise from another unknown crystalline phase. Tilting the diffraction pattern about the [110] direction in Fig. 4(a) approximately 20° produced the [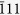] zone axis of α-Fe. The SAED pattern of the [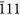]_α_ zone axis and the corresponding bright-field TEM image are shown in Fig. 4(c,d), respectively. In this case, the extra spots cannot be observed, suggesting that the unknown phase possesses a specific orientation with α-Fe. In some early literature, the extra diffraction spots were indexed as ε carbide or a type of cementite^17^,^18^,^19^,^20^. Based on the structure parameters of well-known carbides^30^,^31^, we confirmed that these extra diffraction spots cannot be indexed as any well-known carbide. Taking ε carbide for example, it is well documented that it has the orientation relationship [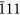]_bcc_// [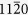]_ε_, (011)_bcc_ // (0001)_ε_^32^,^33^,^34^,^35^,^36^,^37^,^38^. However, when the martensite plate is tilted to the [100] or [111] zone axis, no diffraction spots other than the fundamental bcc diffraction spots were observed. This finding suggests that the extra diffraction spots are not from ε carbide or any other type of carbide.

**Figure 5:**
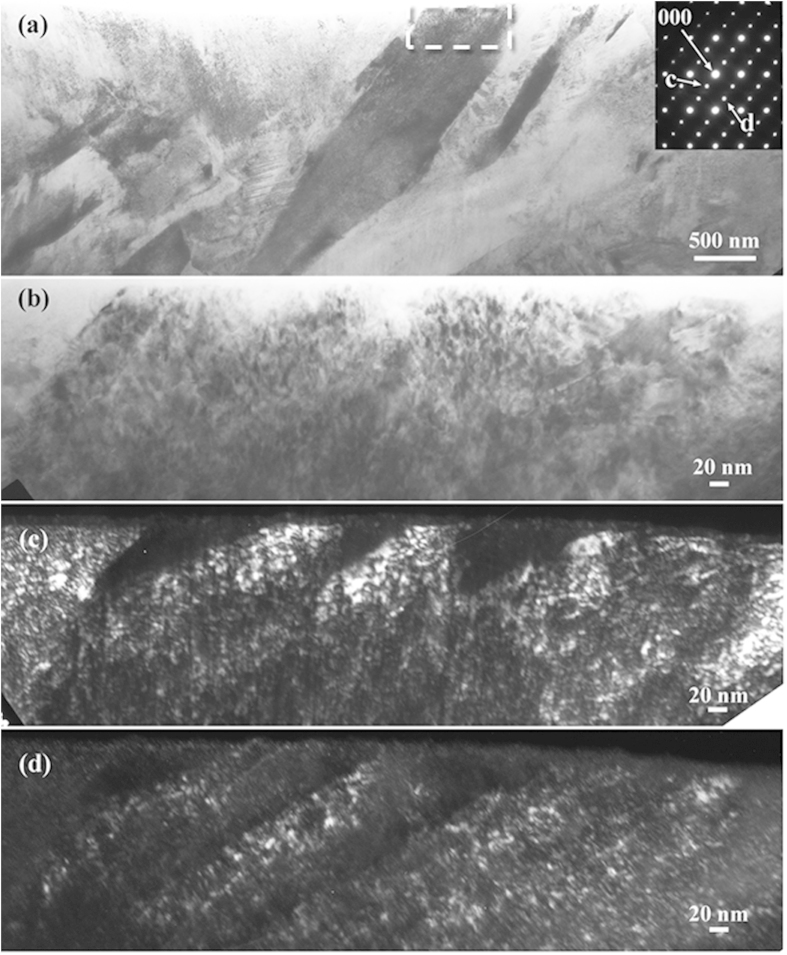
TEM observation results from the as-quenched Fe-0.98 mass% C sample. (**a**) A typical bright-field image of plate martensite and the corresponding SAED pattern observed with the electron beam parallel to [011]_bcc_. (**b**) High magnification of the region outlined by the dashed rectangle in (**a**). (**c,d**) are dark-field images of the diffraction spots *c* and *d* in (**a**), respectively.

To confirm the universality of the existing specific phase that contributes the extra diffraction spots in martensitic carbon steels, an Fe-0.98 mass% C binary alloy was selected for further TEM observations. Figure 5 shows the TEM observation results of the specimen in an as-quenched state. The TEM foil for observation is taken from the middle part of the quenched plate specimen with 0.5 mm in thickness. It can be seen from a typical bright-field TEM micrograph and the corresponding SAED pattern shown in Fig. 5(a) that the observed region contains several martensitic crystal grains with different orientations, and there is no clear evidence of a twin substructure. Figure 5(b) depicts a high-magnification image of the region outlined by the dashed rectangle in Fig. 5(a). It should be noted that unambiguous contrast at the nanometre scale was observed, suggesting that the plate martensite seemingly has an extra substructure rather than a homogenous single phase. To verify the substructure, an intensive diffractometric analysis was performed. Figure 5(c,d) depict the dark-field images by the extra diffraction spots denoted as *c* and *d* shown in Fig. 5(a), respectively. The dark-field images reveal clearly that a high density of ultrafine particles exists in the plate martensite, and no trace of twinned substructure was directly observed in the present study.

**Figure 6:**
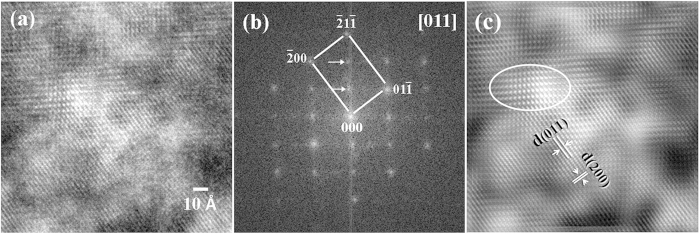
High-resolution TEM lattice image of ultra-fine particles in the plate martensite. (**a**) HRTEM lattice image revealing different lattice contrast. (**b**) Fourier-filtered transformed (FFT) diffraction pattern showing bcc-ferrite and extra spots. (**c**) Inverse FFT image of (**a**) displaying an HRTEM image with bcc and another lattice.

Figure 6(a) shows a high-resolution TEM lattice image of the above ultra-fine particles in the plate martensite. The corresponding Fourier-filtered transformed (FFT) diffraction pattern and the inversed FFT lattice image are shown in Fig. 6(b,c), respectively. Figure 6(b) clearly indicates that the diffraction pattern is composed of two sets of sub-patterns wherein one set is indexed as bcc-Fe or ferrite phase. Considering the fact that no twinning contrast could be observed in the region from the bright, dark and high-resolution TEM observations, the other set of patterns at 1/3(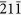)_bcc_ and 2/3(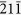)_bcc_, indicated by white arrows in Fig. 6(b), could be ascribed to another specific phase. Again, calculations using parameters of carbides do not achieve a similar result to that shown in Fig. 6(b). From the inverse FFT high-resolution TEM image shown in Fig. 6(c), one can clearly observe local regions with different lattice contrasts, as outlined by the white circle. Obviously, this nanometre-sized region can be attributed only to a specific phase.

Researchers have employed another approach to interpreting the extra spots in diffraction patterns. It is well known that groups of martensite variants form in a single austenite grain during martensitic transformation to accommodate the huge transformation strain. Therefore, there must be pairs of martensite variants with a twin-orientation relationship within a single austenite grain^39^,^40^. If some martensitic variants are sufficiently thin, their diffraction spots in the reciprocal space may elongate and produce high-order Laue spots if the elongated spots are sufficiently long to intersect the Ewald sphere. Twin-effect-induced high-order Laue spots have been confirmed in some materials^41^. However, our calculation shows that the streaking of reciprocal spots does not produce the same diffraction pattern—for example, the pattern shown in Fig. 4(a).

**Figure 7:**
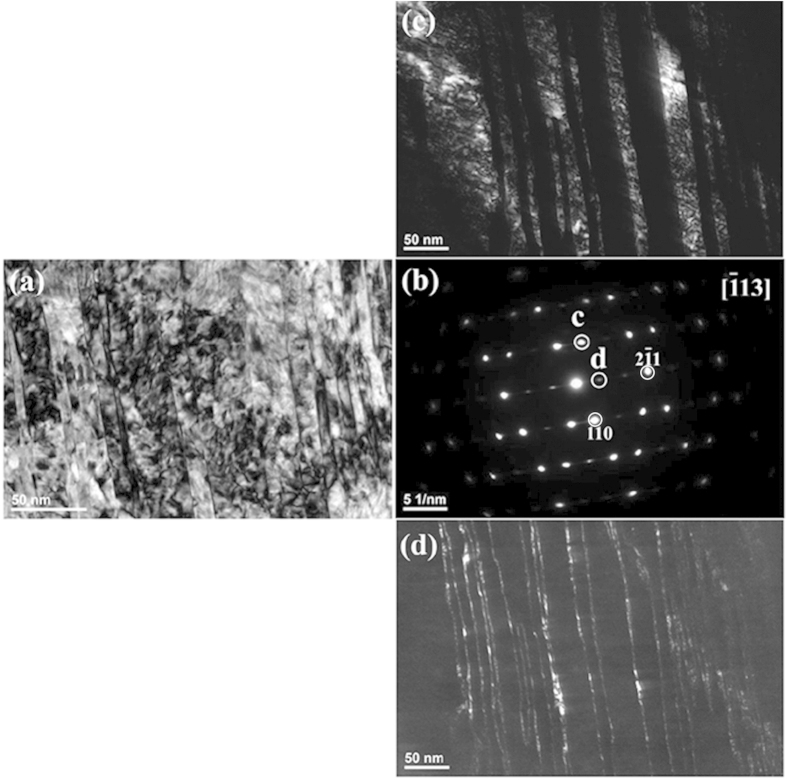
SAED patterns of [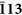]_α_ and the corresponding bright-field TEM images of the as-quenched Fe-0.58 mass% C sample. (**a**) The bright-field image; (**b**) the SAED pattern from α-Fe with [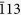]_α_ as the zone axis. (**c,d**) are the dark-field images taken from the spots shown in (**b**) indicated by circles “c” and “d”, respectively.

Furthermore, the subsequent dark-field image observation does not suggest that the extra spots result from merely martensite variants themselves. In Fig. 5, the dark-field images show that the extra spots result from a crystal region several or dozens of nanometres in size. Until now, there has been no report or theoretical analysis claiming that martensite contains substructure at the nanometre scale. The same is true for twinning-structured martensite. Figure 7 shows a typical bright-field TEM image of a twinned region in the as-quenched Fe-0.58 mass% C sample. The corresponding SAED pattern with twinning structure is shown in Fig. 7(b). Figure 7(c,d) are the dark-field images using the diffraction spots “c” and “d” shown in Fig. 7(b), respectively. One can find many bright dots at the nanometre scale scattered along the twin boundary from the dark-field image shown in Fig. 7(d). To the knowledge of the present authors, previous research on conventional martensitic transformation did not predict or consider the presence of these nano-sized regions in Fig. 7(d) or the nano-sized regions in Fig. 5.

## Discussion

Many experimental investigations have demonstrated that a metastable phase, designated as ω, typically precipitates from the matrix of bcc metals and alloys when the bcc lattice becomes unstable by doping, high-speed impact or high pressure^22^,^42^,^43^,^44^,^45^. Omega is a hexagonal structure with a space group of P6/mmm (191), which is different from that of a hexagonal close-packed structure, P6_3_/mmc (194). Metastable ω phase is a common phase in Group IV metals and their alloys^22^,^23^,^24^, such as β-type Ti (Zr or Hf) alloys, heavily deformed pure Mo^46^, β-brass^47^,^48^, Ta^49^ and some Fe-based high alloys^50^,^51^. The ω phase is always coherent with the bcc matrix with the lattice relationships of *a*_ω_ = √2 × *a*_bcc_ and *c*_ω_ = √3/2 × *a*_bcc_, following the orientation relationships of [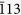]_bcc_//[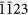]_ω_, (110)_bcc_ // (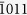)_ω_, and (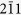) _bcc_ // (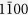)_ω_. According to the mechanism for ω phase formation, the lattice can be obtained by collapsing one pair of (111)_bcc_ planes and keeping the neighbouring (111)_bcc_ plane unaltered within the bcc lattice^49^,^52^.

**Figure 8:**
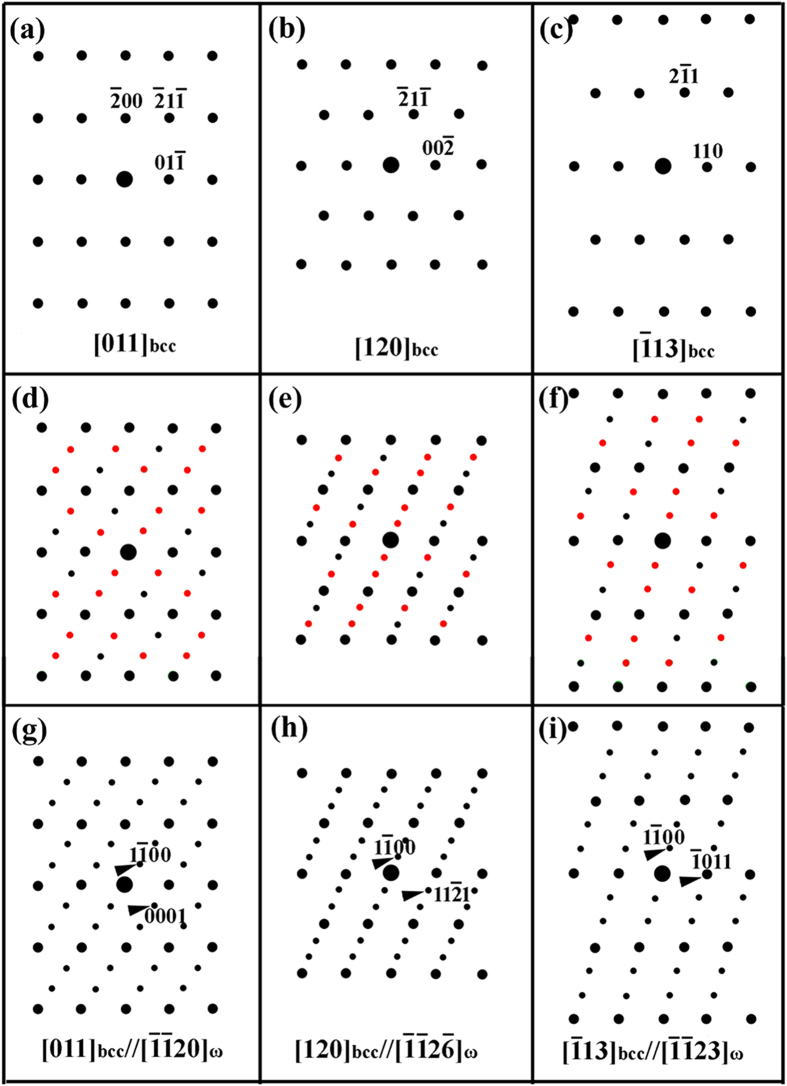
Schematic illustrations of the electron diffraction patterns with the electron beam parallel to the (a) [011], (b) [120] and (c) [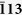] zone axes of a bcc single crystal observed on TEM. In (**d–f**), there are extra sets of diffraction spots in addition to the diffraction spots from the bcc crystal. The small black spots, including those overlapped with the larger black spots from the bcc crystal, have a {112}<111>-type twinning relationship in the bcc crystals. Other small extra spots in red (colour online) were conventionally understood to be double-diffraction spots between matrix and twin. (**g–i**) are rebuilt by using ω phase parameter, where the diffraction spots are exactly the same as in (**d–f**).

Some researchers recently studied the phase-transition pathway by first-principles calculations, showing that the ω structure appears during the fcc-bcc transformation^25^ and the phase transformation follows fcc-ω-bcc or fcc-ω+bcc^25^,^26^. Based on this evidence, it might be reasonable to suppose the existence of the ω phase in carbon steels. We recalculated the electron diffraction patterns of the bcc matrix by taking into account the ω phase. Figure 8 shows schematic illustrations of the electron diffraction patterns with the electron beam parallel to the [011]_bcc_ (Fig. 8(a)), [120]_bcc_ (Fig. 8(b)) and [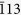]_bcc_ (Fig. 8(c)) zone axes. Figure 8(d) through (f) show that there are extra sets of diffraction spots along with the diffraction spots from the bcc crystal. The small black spots—including those overlapping the larger black spots from the bcc crystal—have a {112}<111>-type twinning relationship in bcc crystals. In addition, some extra small diffraction spots are highlighted in red (colour online). These extra spots were conventionally considered to be double-diffraction spots between the matrix and twin. In fact, if the ω phase is included in the calculation, the corresponding results could be rebuilt into Fig. 8(d) through (f) and the final diffraction patterns could be represented as Fig. 8(g) through (i). The calculated diffraction spots perfectly match the spots observed in 0.58% C and 0.98% C steels, with reference to Figs 1 and 4, 5, 6. For example, the TEM morphologies of the quenched 0.98% C specimen shown in Fig. 5 could be considered important evidence that the extra diffraction spots are not from the double diffraction of twinning but, rather, from a specific phase.

Very recently, an ω-lattice mechanism was proposed to explain the formation of {112}<111> twinning from nanoscale metastable ω precursors^53^, providing a helpful perspective for understanding the results achieved in the present study. On the basis of the proposed ω-lattice mechanism, the {112}<111>-type twins nucleate inside nanoscale ω particles and grow out into bcc matrix. Therefore, the ω phase might act as a precursor phase prior to the formation of martensitic twins. The curved and overlapped twin boundary (Fig. 1) could be closely associated with the ω phase on the twin boundary (see Fig. 1 in ref. 53). Thus, the ω phase might play a crucial role in the martensitic transformation, and the ω lattice mechanism could be helpful to the development of the mechanism for martensitic transformation in steels.

Yonemura *et al.*^54^ investigated the structural change of Fe-C steels when quenched (at approximately 100 K/s) from the molten state by using a time-resolved X-ray diffraction technique with intense synchrotron radiation; they found that fcc-to-bcc phase transformation occurs at a temperature far above the conventionally acknowledged M_s_ temperature. Sherby *et al.*^55^ proposed that a martensitic transformation in carbon steels might take place in two steps—i.e., the formation of a primary martensite and secondary martensite. The primary martensite forms following a sequence in which the fcc austenite first transforms to a hexagonal structure and then to bcc martensite plus C-rich phase. In addition, a “pre-martensite” formed above nominal M_s_ was also proposed by Cayron^56^, who believes that during martensitic transformation, the fcc matrix first transforms to a hexagonal close-packed phase and then to a bcc product^56^. In the present study, we demonstrate that the intermediate phase is in a P6/mmm hexagonal structure rather than a P6_3_/mmc hexagonal close-packed structured phase. The recent first-principles calculation and crystal structure analysis results added further weight to the proposal^25^,^26^. The confirmation of the ω phase in carbon steels appears to be helpful to explicating the physical mechanism for the martensitic transformation in steels and to further improving the design of advanced martensitic steels.

In summary, an intensive TEM investigation has indicated that the extra diffraction spots frequently observed in martensitic carbon steels—which were historically ascribed to the diffraction from either internal twins or carbides—can be attributed to the ω phase, a metastable phase in particle-like morphology with overall size at the nanometre scale. The ω phase, with a hexagonal structure (P6/mmm) of *a*_ω_ = √2 × *a*_bcc_, *c*_ω_ = √3/2 × *a*_bcc_, possesses strict orientation relationships with matrix ferrite as follows: [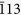]_bcc_ //[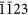]_ω_, (110)_bcc_ //(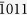)_ω_, (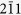)_bcc_ // (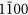)_ω_, which are in agreement with the relationships observed in other bcc metals and alloys.

## Methods

Specimens 0.5 mm thick were cut from commercial carbon-steel bars with 0.58% C and 0.98% C (mass). The detailed chemical compositions of the specimens and their calculated martensitic transformation starting temperatures (M_s_) are given in Table 1. All specimens were sealed in a quartz tube under an Ar atmosphere and austenitized for 30 min at 1373 K before quenching into brine. Specimens for TEM observation were prepared in a conventional way and finished by electropolishing in a twin-jet electropolisher with a chemical solution of 10% HClO_4_ and 90% ethanol at 253 K. The microstructural observation was made with a JEM 2000FX TEM operated at 200 kV and a JEM 2100F high-resolution TEM operated at 200 kV. Electron diffraction patterns of the {112}<111>-type twin structure were calculated using the software application xHREM (HREM Research Inc.).

## Additional Information

**How to cite this article**: Liu, T. *et al.* A new nanoscale metastable iron phase in carbon steels. *Sci. Rep.* **5**, 15331; doi: 10.1038/srep15331 (2015).

## Figures and Tables

**Table 1:** Chemical composition (mass%) and the calculated M_s_ temperatures^57^,^58^ of the steels investigated.

C	Si	Mn	Cr	Fe	M_s_
0.58	1.60	0.58	1.10	bal.	521 K
0.98	0.25	0.33	1.50	bal.	406 K
